# Association between oxidative balance score and psychological disorders in adults

**DOI:** 10.1038/s41598-026-46301-7

**Published:** 2026-06-12

**Authors:** Glareh Koochakpoor, Asma Salari-Moghaddam, Mohammadreza Bagheri, Awat Feizi, Tiffany K Gill, Parisa Hajihashemi, Alireza Ani, Hamidreza Roohafza, Peyman Adibi

**Affiliations:** 1 Maragheh University of Medical Sciences, Maragheh, Iran; 2 Non-Communicable Diseases Research Center, Ilam University of Medical Sciences, Ilam, Iran; 3 Student Research Committee, Ilam University of Medical Sciences, Ilam, Iran; 4 Department of Public Health, Faculty of Health, Ilam University of Medical Sciences, Ilam, Iran; 5 Department of Nursing, School of Nursing and Midwifery, Ilam University of Medical Sciences, Ilam, Iran; 6 Department of Epidemiology and Biostatistics, School of Health, and Clinical Toxicology Research Center, Isfahan University of Medical Sciences, Isfahan, Iran; 7 Adelaide Medical School, Faculty of Health and Medical Sciences, The University of Adelaide, Adelaide, SA 5005 Australia; 8 Nutrition and Food Security Research Center, Isfahan University of Medical Sciences, Isfahan, Iran; 9 Department of Bioinformatics, Isfahan University of Medical Sciences, Isfahan, Iran; 10 Cardiac Rehabilitation Research Center, Cardiovascular Research Institute, Isfahan University of Medical Sciences, Isfahan, Iran; 11 Isfahan Gastroenterology and Hepatology Research Center, Isfahan University of Medical Sciences, Isfahan, Iran

**Keywords:** Depression, Anxiety, Oxidative balance score, OBS, Diseases, Health care, Medical research, Psychology, Psychology, Risk factors

## Abstract

Maintaining a favorable balance between antioxidants and pro-oxidants, rather than merely increasing antioxidant intake, may be critical for mental health. This study examined the association between Oxidative Balance Score (OBS), an integrated measure of both antioxidant and pro-oxidant exposures, and odds of psychological disorders. This cross-sectional analysis used baseline data from the Isfahan Functional Disorders (ISFUN) cohort. Dietary intakes were assessed using a self-administered, Willett-format, Dish-based, 106-item Semi-quantitative Food Frequency Questionnaire (DS-FFQ). OBS was calculated based on 16 dietary and 4 lifestyle factors. Depression and anxiety symptoms were evaluated using the Iranian-validated Hospital Anxiety and Depression Scale (HADS). After adjusting for confounding variables, higher OBS was significantly associated with reduced odds of depression (OR: 0.65; 95% CI: 0.49–0.86) and anxiety (OR: 0.70; 95% CI: 0.53–0.92). In sex-stratified analyses, a significant inverse association was found between OBS with depression (OR: 0.65; 95% CI: 0.45–0.93) and anxiety (OR: 0.64; 95% CI: 0.45–0.91) among women. However, there was no significant association between OBS with depression and anxiety among men. In conclusion, a higher OBS was associated with reduced odds of depression and anxiety in this population, with this inverse association being statistically significant only in women, highlighting the potential importance of a balanced antioxidant-pro-oxidant profile for mental health. Prospective studies are needed to confirm these findings.

## Introduction

Depression, anxiety, and psychological distress are among the most prevalent psychiatric conditions, profoundly affecting emotional regulation and behavioral control^1,2^. In 2008, the World Health Organization (WHO) identified Major Depressive Disorder (MDD) as the third leading cause of global disease burden and projected it to become the foremost contributor by 2030^3^. The pathophysiology of depression involves a complex interplay of cellular and molecular mechanisms, including heightened stress reactivity, neuroinflammation, disrupted neurotransmitter signaling, and impairments in neurogenesis and synaptic plasticity^4^. These processes are often exacerbated by oxidative stress, defined as an imbalance between the production of reactive oxygen species (ROS) and the body’s antioxidant defense systems^5^.

Due to the brain’s exceptionally high metabolic rate-accounting for nearly 20% of total oxygen consumption-the central nervous system (CNS) is particularly susceptible to oxidative damage^6,7^. This vulnerability is further amplified by several factors. Neurons are post-mitotic cells with limited regenerative capacity^8^; neuronal membranes are rich in polyunsaturated fatty acids that are prone to lipid peroxidation; and key neurotransmitters such as dopamine, norepinephrine, and serotonin are inherently unstable and capable of generating additional reactive species through autoxidation^9^.

Despite the well-established involvement of oxidative stress in psychiatric disorders, evidence regarding the relationship between individual antioxidants or pro-oxidants and psychological distress remains inconsistent^10–12^. Most previous studies have examined antioxidant components in isolation without considering the combined effects of various dietary and lifestyle factors. However, an individual’s diet and lifestyle encompass numerous components with both antioxidant and pro-oxidant properties that may exert synergistic or antagonistic effects. In this context, higher antioxidant intake does not necessarily confer greater benefit; rather, maintaining an optimal balance between exposure to dietary and non-dietary antioxidants and pro-oxidants is likely to be more important for mental health.

The Oxidative Balance Score (OBS) is an integrative index that reflects the cumulative influence of both antioxidant (e.g., dietary antioxidants and physical activity) and pro-oxidant (e.g., dietary pro-oxidants, obesity, alcohol use, and smoking) exposures. Consequently, a higher OBS represents a lifestyle favoring a net antioxidant status, indicating the predominance of protective factors over damaging ones^13^. In two previous cross-sectional studies, OBS showed an inverse association with depression, with this association being particularly pronounced in women^14,15^. Given that no study has yet been conducted on the relationship between OBS and psychological disorders including depression and anxiety in Iran, the present study aims to investigate the association between OBS and odds of psychological disorders.

## Methods and materials

### Study population

This cross-sectional study utilized baseline data collected for the Isfahan Functional Disorders (ISFUN) cohort study. The ISFUN study was initiated in 2017 to explore the epidemiology, risk factors, course and prognosis of functional somatic syndromes (FSSs) among a sample of Iranian adults in Isfahan province. The methodology of ISFUN has been extensively described elsewhere^16^. In summary, a total of 1,930 seemingly healthy adults, aged between 18 and 65 years, were recruited from the Kerdabad neighborhood of Isfahan using a census-based sampling method. At baseline and during subsequent annual visits, physical examinations, anthropometric, phenotypic, and biological assessments were performed. Data on functional symptoms, psychological assessments, quality of life, as well as sociodemographic and lifestyle factors were gathered by self-administered questionnaires at baseline and during follow-ups. After excluding those with incomplete data, 1795 participants were analyzed in this study. All participants provided written informed consent prior to their participation in the study. The ISFUN study protocol received approval from both the National Research Ethics Committee of the Iranian Ministry of Health and Medical Education and the Research Ethics Committee of Isfahan University of Medical Sciences (IR.MUI.REC.1395.1.149).

### Dietary intakes assessment

To obtain information regarding the habitual dietary intakes of participants, a self-administered, Willett-format, Dish-based, 106-item Semi-quantitative Food Frequency Questionnaire (DS-FFQ) was applied. This questionnaire was specifically designed and validated for Iranian adults. Additional details regarding the design, foods, and the validity of this questionnaire has been reported elsewhere^17^. Participants were asked to report the amount and frequency of consumption for each food item over the past year, indicating daily (e.g., bread), weekly (e.g., rice, meat), or monthly (e.g., fish) consumption patterns. Household measures were applied to convert portion sizes of consumed foods into grams. Nutrient analysis of diets was performed using a modified version of Nutritionist IV software for Iranian foods (Version 7.0; N-Squared Computing, Salem, OR, USA). Ultimately, the daily intakes of all food items were calculated and utilized in the statistical analyses.

###  OBS estimation

 OBS was calculated using the method described by Goodman et al.^18^. In this study, OBS was determined based on a combination of 16 dietary and 4 lifestyle factors, which included 5 pro-oxidants and 15 antioxidant components. The components divided into four categories: 1) dietary pro-oxidants including total fat and iron, 2) non-dietary pro-oxidants including alcohol use, smoking, and obesity, 3) dietary anti-oxidants including dietary fiber, folic acid, vitamin B6, vitamin B12, vitamin C, vitamin E, niacin, riboflavin, calcium, selenium, copper, magnesium, zinc, and beta-carotene, and 4) non-dietary anti-oxidants including physical activity **(**Table 1**)**. First, dietary components were energy-adjusted before calculating the OBS and were then categorized into tertiles. Antioxidants were scored 0, 1, and 2 for tertiles 1, 2, and 3, respectively, whereas pro-oxidants were scored in the reverse order. For obesity, we assigned 0 if BMI ≥ 30 kg/m^2^ and WC ≥ 102 cm in men or ≥ 88 cm in women, 1 if either BMI ≥ 30 kg/m^2^ or WC ≥ 102 cm in men or ≥ 88 cm in women, and 2 if BMI < 30 kg/m^2^ and WC < 102 cm in men or < 88 cm in women. For smoking, it was assigned 0 for current smokers, 1 for former smokers, and 2 for never smokers. For alcohol use, it was assigned 0 for current use, 1 for former use, and 2 for never use. In terms of physical activity, the score of 0 was assigned for low, 1 for medium, and 2 for high physical activity. The scores of the four components were then summed to obtain the total OBS for each participant. A higher OBS score reflects a greater overall antioxidant capacity.

**Table 1:** Oxidative balance score components.

OBS components	Assignment scheme
Non-dietary pro-oxidants	
Obesity	0 = BMI≥30 kg/m^2^ and WC ≥ 102 cm in men or ≥ 88 cm in women 1 = BMI ≥ 30 kg/m^2^ or WC ≥ 102 cm in men or ≥ 88 cm in women 2 = BMI < 30 kg/m^2^ and WC < 102 cm in men or < 88 cm in women
Smoking	0 = current smoker, 1 = former smoker, 2 = never smoker
Alcohol use	0 = current use, 1 = former use, 2 = never use
Non-dietary anti-oxidant	
Physical activity (MET-min/week)	0 = low (1st tertile), 1 = medium (2nd tertile), 2 = high (last tertile)
Dietary pro-oxidants	
Total fat (g/d)	0 = high (3rd tertile), 1 = medium (2nd tertile), 2 = low (1st tertile)
Iron (mg/d)	0 = high (3rd tertile), 1 = medium (2nd tertile), 2 = low (1st tertile)
Dietary anti-oxidants	
Dietary fiber (g/d)	0 = low (1st tertile), 1 = medium (2nd tertile), 2 = high (last tertile)
Folic acid (µg/d)	0 = low (1st tertile), 1 = medium (2nd tertile), 2 = high (last tertile)
Vitamin B6 (mg/d)	0 = low (1st tertile), 1 = medium (2nd tertile), 2 = high (last tertile)
Vitamin B12 (µg/d)	0 = low (1st tertile), 1 = medium (2nd tertile), 2 = high (last tertile)
Vitamin C (mg/d)	0 = low (1st tertile), 1 = medium (2nd tertile), 2 = high (last tertile)
Vitamin E (mg/d)	0 = low (1st tertile), 1 = medium (2nd tertile), 2 = high (last tertile)
Niacin (mg/d)	0 = low (1st tertile), 1 = medium (2nd tertile), 2 = high (last tertile)
Riboflavin (mg/d)	0 = low (1st tertile), 1 = medium (2nd tertile), 2 = high (last tertile)
Calcium (mg/d)	0 = low (1st tertile), 1 = medium (2nd tertile), 2 = high (last tertile)
Selenium (µg/d)	0 = low (1st tertile), 1 = medium (2nd tertile), 2 = high (last tertile)
Copper (mg/d)	0 = low (1st tertile), 1 = medium (2nd tertile), 2 = high (last tertile)
Magnesium (mg/d)	0 = low (1st tertile), 1 = medium (2nd tertile), 2 = high (last tertile)
Zinc (mg/d)	0 = low (1st tertile), 1 = medium (2nd tertile), 2 = high (last tertile)
β-Carotene (µg/d)	0 = low (1st tertile), 1 = medium (2nd tertile), 2 = high (last tertile)

OBS: oxidative balance score; BMI: body mass index; WC: waist circumference; MET: metabolic equivalent of task.

### Depression and anxiety symptoms assessment

Depression and anxiety symptoms were assessed using an Iranian-validated version of the Hospital Anxiety and Depression Scale (HADS) questionnaire^19,20^. This 14-item questionnaire is divided into two sections, each evaluating the severity of anxiety and depression. Each section contains 7 items with a 4-point rating scale, resulting in total scores that range from 0 (indicating no depression or anxiety) to 21 (indicating severe depression or anxiety). Scores of ≤ 7 in each section were considered to indicate normal status, while scores of ≥ 8 were considered to represent the presence of depression or anxiety in this study. A study involving 167 Iranian adults demonstrated that the questionnaire provides relatively valid measures of mental health^20^.

### Assessment of other variables

Trained personnel adhered to standardized protocols for the collection of anthropometric measurements. Height was recorded to the nearest 0.5 cm with a nonelastic measuring tape while the participant stood barefoot and in a relaxed posture. Weight was assessed on a scale to the nearest 100 g, with participants dressed in light clothing. Body mass index (BMI) was calculated by dividing weight (in kilograms) by height (in meters squared). Physical activity was evaluated using the International Physical Activity Questionnaire (IPAQ) and reported as metabolic equivalent (MET) minutes per week^21^.

### Statistical analysis

Participants were categorized into tertiles according to the distribution of OBS in the study population. General characteristics of study participants across tertiles of OBS were presented as means ± SDs for continuous variables and percentages for categorical variables. To examine the differences across tertiles, we used ANOVA for continuous variables and chi-square test for categorical variables. We used binary logistic regression to estimate ORs and 95% CIs for the presence of depression and anxiety across tertiles of OBS in crude and multivariable-adjusted models. The trend of ORs across tertiles of OBS was determined by considering tertiles of OBS as ordinal variables in the logistic regression analysis. In these analyses, age and sex (male/female) were adjusted in the first model. Second model was additionally adjusted for socioeconomic status (low/medium/high), education (0–5 y/6–12 y/>12 y), marital status (married/un-married), sleep quality (very good/moderate good/moderate bad/very bad), and antidepressant use (yes/no). All statistical analyses were done using the Statistical Package for Social Sciences (version 20; SPSS Inc.). *P* < 0.05 was considered as statistically significant.

## Results

In the whole population, the prevalence of depression and anxiety were 33.9% (*n* = 609) and 35.9% (*n* = 646), respectively. The mean age of study participants was 39.63 ± 10.2 years.

General characteristics of study participants across tertiles of OBS are shown in Table 2. Compared with those in the lowest tertile of OBS, those in the highest tertile were less likely to be younger, and have depression and anxiety, and more likely to be female, physically active, and had lower WC. No other significant differences were observed in terms of other variables across tertiles of OBS.

**Table 2:** General characteristics of study participants across tertiles of OBS^a^.

Tertiles of OBS				
Variables	T_1_	T_2_	T_3_	*P*-value^b^
Subjects, n	550	619	626	
Age, y	38.6 ± 10.04	39.98 ± 10.23	40.2 ± 10.2	0.02
BMI, kg/m^2^	27.18 ± 5.0	27.25 ± 5.2	26.8 ± 4.5	0.20
WC, cm	92.1 ± 12.2	90.9 ± 12.3	89.4 ± 10.7	< 0.001
Physical activity, MET-min/week	2605.9 ± 2987.2	2633.4 ± 2981.4	2999.4 ± 3099.1	0.04
Female, %	47.5	57.5	60.5	< 0.001
Married, %	82.4	82.7	83.2	0.92
Current smokers, %	26.7	19.5	11.6	0.23
Antidepressant use, %	6.7	7.1	6.5	0.92
Socioeconomic status, %				0.052
Low	13.7	16.5	11.6	
Medium	54.7	57.8	57	
High	31.6	25.6	31.4	
Education, %				0.14
0–5 y	10.4	15.0	14.3	
6–12 y	52.9	52.4	52.6	
>12 y	36.6	32.5	33.1	
Sleep quality, %				0.80
Very good	17.6	19.4	20	
Moderate good	61.5	61.6	61.0	
Moderate bad	14.4	14.2	12.9	
Very bad	6.5	4.8	6.1	
Depression, %	38	33.6	30.7	0.02
Anxiety, %	40.2	34.4	33.9	0.04

^a^Data are percentage or mean ± standard deviation (SD).

^b^Obtained from ANOVA for continuous variables or chi-square test for categorical variables.

Dietary intakes of study participants across tertiles of OBS are shown in Table 3. Compared to participants in the bottom tertile, those in the top tertile of OBS had higher intakes of dietary fiber, total fat, folic acid, vitamin B6, vitamin B12, vitamin C, vitamin E, riboflavin, calcium, iron, selenium, copper, magnesium, zinc, and β-Carotene and lower intakes of energy and niacin.

**Table 3:** Dietary intakes of study participants across tertiles of OBS^a^.

Tertiles of OBS				
Variables	T_1_	T_2_	T_3_	*P*-value^b^
Subjects, n	615	594	586	
Energy (kcal/d)	2537.1 ± 795.01	2165.1 ± 759.7	2191.3 ± 752.5	< 0.001
Dietary fiber (g/d)	13.65 ± 3.2	15.41 ± 3.5	18.53 ± 4.09	< 0.001
Total fat (g/d)	94.8 ± 35.8	106.8 ± 24.2	106.1 ± 18.3	< 0.001
Folic acid (µg/d)	215.5 ± 73.2	289.3 ± 72.1	355.2 ± 90.7	< 0.001
Vitamin B6 (mg/d)	1.19 ± 0.77	1.53 ± 0.51	1.82 ± 0.40	< 0.001
Vitamin B12 (µg/d)	2.90 ± 4.6	5.80 ± 4.50	6.79 ± 3.77	< 0.001
Vitamin C (mg/d)	76.8 ± 35.2	109.7 ± 43.9	157.4 ± 65.1	< 0.001
Vitamin E (mg/d)	1.08 ± 0.99	1.56 ± 0.94	2.06 ± 0.91	< 0.001
Niacin (mg/d)	23.59 ± 4.8	22.5 ± 5.02	21.9 ± 5.00	< 0.001
Riboflavin (mg/d)	1.26 ± 0.47	1.69 ± 0.81	1.98 ± 0.95	< 0.001
Calcium (mg/d)	708.8 ± 242.3	826.2 ± 328.8	954.9 ± 321.3	< 0.001
Iron (mg/d)	21.85 ± 9.1	22.7 ± 8.7	26.1 ± 13.8	< 0.001
Selenium (µg/d)	0.01 ± 0.01	0.014 ± 0.01	0.02 ± 0.01	< 0.001
Copper (mg/d)	1.01 ± 0.51	1.41 ± 1.38	1.65 ± 1.65	< 0.001
Magnesium (mg/d)	193.8 ± 47.1	237.5 ± 36.9	272.05 ± 43.1	< 0.001
Zinc (mg/d)	8.37 ± 3.27	10.39 ± 2.94	11.4 ± 2.53	< 0.001
β-Carotene (µg/d)	212.1 ± 123.6	320.3 ± 169.4	460.7 ± 250.3	< 0.001

^a^Data are mean± standard deviation (SD).

^b^Obtained from ANOVA.

Crude and multivariable adjusted ORs and 95% CIs for depression and anxiety across tertiles of OBS are shown in Table 4. In the crude model, greater OBS was associated with lower odds of depression (OR: 0.72; 95% CI: 0.56–0.91). This association remained significant after adjustment for potential confounders; such that those with higher OBS had 35% lower chance for depression (OR: 0.65; 95% CI: 0.49–0.86). Regarding anxiety, a significant inverse association was found between OBS and anxiety in the fully adjusted model (OR: 0.70; 95% CI: 0.53–0.92).

**Table 4:** Odds ratios and 95% CIs for psychological disorders across tertiles of OBS^a^.

	Tertiles of OBS			*P*-trend
	T_1_	T_2_	T_3_	
Depression				
Cases, n	209	208	192	
Crude	1.00	0.82 (0.65–1.04)	0.72 (0.56–0.91)	0.008
Model I^b^	1.00	0.76 (0.59–0.97)	0.65 (0.51–0.83)	0.001
Model II^c^	1.00	0.72 (0.55–0.95)	0.65 (0.49–0.86)	0.003
Anxiety				
Cases, n	221	213	212	
Crude	1.00	0.78 (0.61–0.99)	0.76 (0.60–0.96)	0.02
Model I^b^	1.00	0.70 (0.55–0.90)	0.67 (0.52–0.86)	0.002
Model II^c^	1.00	0.68 (0.52–0.89)	0.70 (0.53–0.92)	0.01

^a^Data are OR (95% CI).

^b^Model I: adjusted for age and sex.

^c^Model II: additionally, adjusted for socioeconomic status, education, marital status, sleep quality, and antidepressant use.

Gender-stratified crude and multivariable-adjusted ORs and 95% CIs for psychological disorders across tertiles of OBS are provided in Table 5. With regards to depression, women in the highest tertile of OBS had a lower chance for depression compared with those in the lowest tertile (OR: 0.65; 95% CI: 0.45–0.93). Furthermore, we observed a significant inverse association between OBS and anxiety among women (OR: 0.64; 95% CI: 0.45–0.91). In the crude and age-adjusted models, there was a suggestion of an inverse association between OBS and depression among men, but this was attenuated and became non-significant after adjustment for socioeconomic and lifestyle factors in the fully adjusted model (OR: 0.70; 95% CI: 0.45–1.10). We observed no significant association between OBS and anxiety among men (OR: 0.88; 95% CI: 0.56–1.38).

**Table 5:** Sex-stratified odds ratios and 95% CIs for psychological disorders across tertiles of OBS^a^.

	Tertiles of OBS			*P*-trend
	T_1_	T_2_	T_3_	
Men				
Depression				
Cases, n	71	72	76	
Crude	1.00	0.67 (0.46–0.97)	0.64 (0.43–0.93)	0.01
Model I^b^	1.00	0.66 (0.45–0.96)	0.62 (0.42–0.91)	0.01
Model II^c^	1.00	0.56 (0.35–0.87)	0.70 (0.45–1.10)	0.10
Anxiety				
Cases, n	67	69	83	
Crude	1.00	0.78 (0.54–1.13)	0.75 (0.51–1.02)	0.13
Model I^b^	1.00	0.77 (0.53–1.13)	0.72 (0.49–1.07)	0.10
Model II^c^	1.00	0.68 (0.48–1.04)	0.88 (0.56–1.38)	0.34
Women				
Depression				
Cases, n	169	126	95	
Crude	1.00	0.86 (0.62–1.19)	0.69 (0.50–0.96)	0.02
Model I^b^	1.00	0.84 (0.60–1.16)	0.68 (0.49–0.95)	0.02
Model II^c^	1.00	0.85 (0.59–1.22)	0.65 (0.45–0.93)	0.01
Anxiety				
Cases, n	171	136	120	
Crude	1.00	0.67 (0.48–0.92)	0.64 (0.46–0.88)	0.009
Model I^b^	1.00	0.65 (0.47–0.90)	0.63 (0.46–0.87)	0.008
Model II^c^	1.00	0.68 (0.48–0.98)	0.64 (0.45–0.91)	0.01

^a^Data are OR (95% CI).

^b^Model I: adjusted for age.

^c^Model II: additionally, adjusted for socioeconomic status, education, marital status, sleep quality, and antidepressant use.

## Discussion

In the present cross-sectional study, we observed a significant inverse association between OBS and odds of depression and anxiety. This association was sex-specific, with higher OBS being associated with reduced odds of depression and anxiety among women. However, we failed to find any significant association between OBS with depression and anxiety among men.

However, the inverse association observed in our study was weaker than that reported in previous studies^14,15,22^. Importantly, our analysis accounted for the use of antidepressant medications, whereas Liu et al.’s study^14^ did not sufficiently consider participants’ use of medications that may influence both oxidative stress and mood, potentially introducing confounding factors. In addition, two other studies reported a stronger inverse association between OBS and depression, one conducted among cancer patients^15^ and the other among post-stroke individuals^22^. However, these findings cannot be directly generalized to the broader population, as stroke survivors and cancer patients often exhibit higher baseline oxidative stress, multiple comorbidities, and lifestyle factors that may independently influence both OBS and mood^23^. Therefore, although the underlying mechanisms linking oxidative stress and mood regulation may overlap, the magnitude of the association observed in post-stroke and cancer populations may not accurately reflect that of the general population.

A higher OBS, reflecting a dietary pattern rich in antioxidants and low in pro-oxidants, may be associated with lower likelihood of depression by multiple biological pathways. First, by enhancing antioxidant defenses, a higher OBS could reduce oxidative stress, which can cause lipid peroxidation, DNA damage, and protein oxidation in neural tissues, leading to neuronal dysfunction and apoptosis^24^. The brain is particularly vulnerable to oxidative damage due to its high oxygen consumption and lipid-rich composition^9^. These oxidative insults may impair synaptic plasticity, reduce neurogenesis (particularly in the hippocampus), and disrupt neurotransmission, all of which are key processes in mood regulation^25^. Second, antioxidant-rich diets may suppress chronic low-grade inflammation, as oxidative stress and inflammation are tightly linked and jointly contribute to neurotoxicity and depressive symptoms^26^. Third, nutrients abundant in high-OBS diets (e.g., B vitamins, magnesium, zinc) act as cofactors in neurotransmitter synthesis and may prevent oxidative degradation of monoamines such as serotonin and dopamine, thereby supporting mood regulation^27,28^. Fourth, a favorable oxidative balance may preserve mitochondrial function and neuronal bioenergetics, as mitochondrial dysfunction is strongly associated with mood disorders and oxidative insult is a major contributor^29^.

To the best of our knowledge, our study is the first to examine the relationship between the OBS and anxiety. Although previous studies have explored the role of individual biomarkers of oxidative damage or antioxidant levels in development of anxiety, their findings have been inconsistent^30,31^. These contradictions highlight the importance of integrating both prooxidants and antioxidants into a single score when studying their relationship with anxiety. This holistic approach provides a more comprehensive assessment of the body’s overall oxidative state, which may explain why a higher OBS is associated with a reduced risk of anxiety.

The inverse association between OBS and anxiety observed in our study may be explained by several potential mechanisms. Improved oxidative balance may help regulate hypothalamic-pituitary-adrenal (HPA) axis, promoting a healthier stress response^32^. Oxidative stress can alter brain function, particularly in regions involved in mood regulation, such as the prefrontal cortex and amygdala^33^. Gamma-aminobutyric acid (GABA) is an inhibitory neurotransmitter that plays a key role in anxiety regulation. Oxidative stress may impair GABA receptors function, leading to heightened anxiety. Conversely, improved oxidative balance may enhance GABAergic function, promoting calmness and reducing anxiety symptoms^34^. Furthermore, improved oxidative balance may attenuate excessive sympathetic nervous system activity, thereby alleviating anxiety symptoms^35^.

The sex-specific association observed in this study, wherein a higher OBS was linked to reduced odds of depression and anxiety only among women, may be partly explained by sex-related biological differences. Estrogen exerts antioxidant and neuroprotective effects, enhancing antioxidant enzyme activity, and reducing oxidative damage in neural tissues^36^. Women also show greater resistance to oxidative stress and mitochondrial dysfunction^37^, which may amplify the benefits of a favorable oxidative balance. Hormonal modulation of neurotransmitter systems and neuroplasticity, particularly in mood-related brain regions, further supports a differential vulnerability to oxidative stress across sexes^38,39^.

This study has several strengths. Notably, it is the first to investigate the relationship between OBS and anxiety. Additional strengths include the large sample size and the consideration of a wide range of potential confounders in the statistical analyses. Nevertheless, some limitations should be acknowledged. The cross-sectional design of this study restricts the ability to infer causality. Therefore, reverse causality must be considered, as individuals with depression and anxiety may have poorer diets and lifestyles, leading to a lower OBS. Such designs do not allow us to determine whether psychological disorders lead to low OBS, or whether low OBS precede the development of psychological disorders; therefore, prospective studies are needed to confirm these findings. While we adjusted for several potential factors, the possibility of residual confounding is inevitable. Additionally, reliance on self-reported data may result in misclassification. The OBS is derived from dietary and lifestyle factors, many of which are self-reported (e.g., using a validated FFQ). As a result, the OBS may not fully capture all relevant pro-oxidant and antioxidant exposures, including unmeasured dietary components or environmental factors.

In conclusion, OBS was strongly inversely associated with depression and anxiety. These findings emphasize the importance of following an antioxidant-rich diet and lifestyle as a potential strategy to prevent psychological disorders.

## Data Availability

The datasets generated and/or analyzed during the current study are not publicly available due to the institution’s policy but are available from the corresponding author on reasonable request.
